# The Impact of eBook Clubs on Apathy Among Long Term Care Residents: A Pilot Study

**DOI:** 10.1177/30495334251345093

**Published:** 2025-06-15

**Authors:** Aderonke Agboji, Shannon Freeman, Davina Banner, Joshua Armstrong, Melinda Martin-Khan

**Affiliations:** 1 University of Northern British Columbia, Prince George, BC, Canada; 2 Lakehead University, Thunder Bay, ON, Canada; 3 Exeter Medical School, University of Exeter, Devon, UK

**Keywords:** nursing home, eReaders, technology, mixed methods, intervention

## Abstract

Apathy, prevalent among long-term care facilities (LTCF) residents, diminishes motivation, social interaction, and quality of life. This study explored the impact of eBook clubs as a non-pharmacological intervention to reduce apathy. A convergent parallel mixed-methods design was employed with 20 residents from four LTCF participating in a 3-month program. Apathy was assessed using the Geriatric Depression Scale (GDS-3A) before and after the intervention, with paired t-tests and Cohen’s d measuring changes. Qualitative insights were derived from semi-structured interviews and thematic analysis. Apathy prevalence dropped from 55% to 35%, and mean scores decreased significantly (1.6–0.9; Cohen’s *d* = 0.85). Participants highlighted cognitive, emotional, and social benefits, valuing program flexibility and eReaders but noting some preference for physical books. These findings suggest eBook clubs as a scalable, cost-effective strategy for LTCF. Future studies should evaluate its broader applicability and explore culturally tailored implementations.

## What This Paper Adds

- Demonstrates the effectiveness of eBook clubs as a non-pharmacological intervention for reducing apathy among LTCF residents.
- Highlights the multidimensional benefits of eBook clubs, including cognitive stimulation, emotional upliftment, and enhanced social interaction, as key mechanisms for improving well-being in older adults.
- Provides evidence of the feasibility and acceptability of eReaders in LTCF, showcasing their adaptability for residents with diverse needs and preferences.

## Applications of Study Findings

- **Gerontological Practice**: eBook clubs offer LTCF a scalable and cost-effective group activity to promote social connection, reduce isolation, and enhance cognitive engagement among residents.
- **Policy**: Findings support investments in digital literacy programs and infrastructure in LTCF, including eReaders and staff training, to expand access to stimulating activities.
- **Research**: Establishes a foundation for future studies on the long-term effects of digital group interventions and comparisons with other non-pharmacological therapies to reduce apathy in aging populations.

## Introduction

Apathy, characterized by a lack of motivation and diminished interest in daily activities, is a significant health concern in long-term care facilities (LTCF) residents (Jao et al., 2015, 2019; Volicer, 2016; Volicer et al., 2013). It affects the cognitive, behavioral, emotional, or social domains (Robert et al., 2018), leading to reduced problem-solving interest, withdrawal from activities, lack of response to motivational cues, or social disengagement (Le Heron et al., 2019; Marin, 1991; Sockeel et al., 2006). Unlike depression, which involves sadness and emotional distress, apathy is marked by emotional flatness and dependence on others for activity initiation, posing unique challenges (Marin et al., 1994). Apathy adversely affects residents by reducing health-related quality of life, accelerating cognitive decline, and increasing mortality risk, while also burdening caregivers due to uncooperative behavior (Jao et al., 2019; Nijsten et al., 2017, 2019).

Pharmacological and non-pharmacological interventions aim to address apathy by encouraging activity participation. Due to trial limitations and side effects of pharmacological treatments (Agboji et al., 2024; Ruthirakuhan et al., 2018; Sepehry et al., 2017), non-pharmacological approaches are preferred (Zuidema et al., 2009). Music therapy, multisensory stimulation, pet therapy, and cognitive stimulation effectively reduce apathy in LTCF residents by providing engaging experiences (Cai et al., 2020; Tan et al., 2022). However, these interventions require significant resources and training, posing challenges for implementation in LTCF (Md Hussin et al., 2023). Additionally, therapeutic and leisure activities reduce apathy by fostering social engagement and reducing loneliness through group interactions (Altintas et al., 2017; Ellis et al., 2016). Personalizing interventions, such as hobbies or familiar activities, enhances engagement, while tailored social activities with appropriate materials support participation despite impairments. Emerging technologies including virtual reality (VR) and companion robots such as PARO offer promising solutions for reducing apathy but face challenges with complexity, side effects, and usability, particularly for residents with cognitive or physical impairments (Moyle et al., 2013; Saredakis et al., 2021). Adapting these technologies is essential to meet LTCF needs.

Reading is a cognitively stimulating activity that can mitigate apathy by promoting mental engagement and emotional well-being. However, challenges such as vision impairment, frailty, and limited access to books often hinder continued reading in later life. Technology-based solutions, specifically, eReaders, have made literature more accessible, encouraging frequent reading and reducing social isolation among older adults (de Oliveira et al., 2020; Gitlow, 2014). Despite these benefits, limited research has explored the impact of group reading sessions facilitated through digital means (eBook clubs) on self-report apathy among LTCF residents. This study addresses this gap by pilot testing the effect of eBook clubs on reducing apathy and integrating quantitative and qualitative findings to better understand the contributing factors.

## Methods

### Study Design

This study used a convergent parallel mixed-methods design (Creswell & Plano Clark, 2018). Quantitative and qualitative data were collected simultaneously to provide a comprehensive understanding of the impact of eBook clubs on apathy among LTCF residents. This design enabled the integration of both objective measures of apathy and subjective experiences of participants, ensuring complementary insights. Following the principles of convergent parallel design, the quantitative and qualitative findings were integrated at the interpretation stage. The quantitative reduction in apathy scores was cross-referenced with themes from the qualitative data to provide a deeper understanding of key factors contributed to the observed improvements. A joint display table was used to present the integration of both data strands (Fetters, 2020).

### Setting and Participants

Participants were recruited from four LTCF located in rural areas. Inclusion criteria required participants to (1) have an interest in reading, (2) be able to read and speak English, (3) have the ability or willingness to use digital technology, and (4) provide informed consent. Residents with severe cognitive or physical impairments that precluded group participation were excluded. A purposive sampling strategy was used to recruit participants. Recruitment efforts included information sessions at each LTCF, posters displayed in common areas, and support from staff (e.g., recreation therapists) in identifying interested residents. Participants were provided with a copy of the information letter 2 weeks prior to enrollment and were assessed for decision-making capacity using the University of California, San Diego Brief Assessment of Capacity to Consent (UBACC; Jeste et al., 2007).

### Study Procedure

The eBook club program was implemented over 12 weeks in each site. Each participant received access to eReaders (KobolibraH20) with a minimum of 20 pre-selected books tailored to their interests, based on preferences identified during the first interview. Books were freely accessible through the Rakuten Kobo platform (www.kobo.com), and participants were encouraged to request additional books throughout the program, with a waiting period of 1 to 2 weeks for new titles. The intervention consisted of weekly group reading sessions facilitated by trained staff and involved 4 to 8 participants per group. Each session lasted 45 to 75 min, depending on participant preferences, and included reading aloud and group discussions about preselected books. This design aimed to foster social engagement, cognitive stimulation, and emotional connection among participants. Independent reading was included as a supplementary activity, providing participants with eReaders and access to a curated library of books. While participants were encouraged to explore the eReaders between sessions, the primary focus remained on group interactions during the weekly meetings. For participants unfamiliar with digital devices, a hands-on tutorial was provided during the orientation session.

### Quantitative Data Collection and Analysis

Quantitative data were collected using the Geriatric Depression Scale (GDS-3A), which includes three apathy-related items. The GDS-3A has been employed in multiple studies (Adams, 2001; Bertens et al., 2017; Grool et al., 2014; Ligthart et al., 2012). The items included the following questions: (1) “Have you dropped many of your activities and interests?” (2) “Do you prefer to stay in your room rather than going out and doing new things?” and (3) “Do you feel full of energy?” For questions 1 and 2, a score of 1 was assigned for a “Yes” response, while for question 3, a score of 0 was assigned for a “Yes” response. The validity of the GDS-3A (Geriatric Depression Scale-3) as a measure of apathy has been explored in two populations of older adults including those with and without depression.

According to Bertens et al. (2017), the GDS-3A shows varying sensitivity and specificity depending on depression status. Among older adults with depression, it had 32.8% sensitivity and 92.6% specificity, indicating high accuracy in ruling out apathy. For those without depression, sensitivity was 29.3%, with specificity at 88.5%. These results suggest the GDS-3 is more effective at confirming the absence of apathy than detecting it, particularly in individuals with co-existing depression. Despite its limitations, the GDS-3A remains a valuable tool for initial screening purposes. The GDS-3A includes key components relevant to apathy detection, making it useful for prompting further clinical investigation (Szymkowicz et al., 2022). Its three-item structure offers psychometric and evaluative advantages over single-item approaches (Szymkowicz et al., 2022). The selection of the GDS-3A was guided by pragmatic and contextual considerations specific to the study setting. Firstly, our research was conducted in long-term care environments where participants exhibited varying degrees of cognitive functioning and fluctuating levels of engagement. In these real-world contexts, the GDS-3A provided a brief, easy-to-administer, and low-burden screening tool suitable for use by researchers without requiring extensive training or certification. Secondly, while we acknowledge that the GDS-3A is not a diagnostic instrument for apathy, it was not used for diagnostic purposes in our study. Instead, it functioned as a proxy measure for observable behavioral disengagement, consistent with its use in prior studies (Adams, 2001; Bertens et al., 2017; Grool et al., 2014; Ligthart et al., 2012; Maruta et al., 2021). Lastly, we were mindful of the GDS-3A’s psychometric limitations and addressed this by incorporating a mixed-methods approach. The qualitative component of the study provided a richer, context-sensitive understanding of participant engagement, emotional responsiveness, and perceived changes related to apathy. This methodological complementarity helped to enhance the depth and interpretive validity of our findings and provided a more nuanced picture than quantitative screening alone could achieve.

In this study, a cut-off score of ≥2 indicated apathy (Bertens et al., 2017). To maintain consistency across sites, the first author administered the GDS-3A pre- and post-intervention, measuring apathy at baseline and after the program. Descriptive and inferential statistics were used to evaluate the eBook club’s impact on self-reported apathy. Demographic data, such as age and gender, were analyzed using Excel for descriptive statistics, providing an overview of the participant sample. Paired t-tests were conducted in SPSS (IBM Corp, 2023) to assess the program’s impact. The analysis emphasized Cohen’s d as a measure of effect size over *p-*values, offering a clearer interpretation of the intervention’s impact, with Cohen’s d values indicating the strength of the observed changes (Cohen, 1988). For analysis, apathy scores were categorized into a binary variable: scores of 2 or higher indicated “apathy,” while scores below 2 were classified as “no apathy.”

### Qualitative Data Collection and Analysis

Qualitative data were collected through semi-structured interviews conducted at enrollment and after the program. The first interview focused on reading habits and expectations, while the second explored participants’ experiences with the eBook club and eReaders. Interviews were audio-recorded, transcribed verbatim, and analyzed using NVivo 12 software (QSR, 2020). Thematic analysis with open coding identified key themes and patterns, ensuring accurate representation of participants’ experiences (Bazeley & Jackson, 2013; Braun & Clarke, 2006). Ethical approval was obtained via the Harmonized Ethics Review and Institutional Research Ethics Board. Informed consent was secured at enrollment and reconfirmed before each session.

### Reflexivity

Reflexivity is vital in mixed-methods research to recognize how researchers’ backgrounds and assumptions can influence the study process (Finlay, 2002). In this study, the first author’s involvement in recruitment, data collection, and analysis posed a potential for bias in data interpretation. To address this, the research team engaged in ongoing self-reflection, remaining open to unexpected findings, whether positive or negative, and critically examining their expectations about the intervention’s effectiveness. Additionally, the team acknowledged that pre-existing relationships between staff and residents might affect participants’ responses. To mitigate this, interviews were conducted in neutral settings by the first and second authors, who were not involved in the residents’ care.

### Credibility and Reliability

To ensure credibility and reliability, we employed triangulation by using multiple data sources including pre- and post-apathy scores and semi-structured interviews to cross-validate findings, enhancing trustworthiness (Creswell & Plano Clark, 2018). Transcripts were independently coded by the first and second authors using NVivo 12, with discrepancies resolved through discussion and consensus with the third author. Regular peer debriefing sessions were conducted throughout the coding process, allowing the research team to challenge assumptions and explore alternative interpretations, reducing the risk of confirmation bias (Creswell & Poth, 2018).

## Results

### Sample Characteristics

Table 1 shows the characteristics of the study participants. A total of 20 residents participated fully in the program. The mean age of participants was 76.4 years (*SD* = 7.2) with a range between 51 and 98 years. The majority identified as woman (75%), half of the participants (50%) completed high school, College or University degree, and over half were widowed (60%). Less than half (40%) reported that they had never used technology before but were excited to try out a technology for reading. Many participants described themselves as avid readers, engaging with a variety of texts for a considerable portion of each day and had experienced a change in their reading habits since admission into LTCF (25% read less and 30% read more). Fictional books were of interest to most of the participants (80%) and none of the participants had a dementia diagnosis.

**Table 1.** Sample Characteristics.

Characteristics	% (*n*)
Age (years)	
50–60	15 (3)
61–70	10 (2)
71–80	30 (6)
81–90	35 (7)
90+	10 (2)
Gender	
Man	25 (5)
Woman	75 (15)
Other	0
Marital status	
Married	15 (3)
Never married	15 (3)
Widowed/separated/divorced	70 (14)
Education	
College/University	20 (4)
High school	30 (6)
Grade 7–9	45 (9)
Grade 1 < 6	5 (1)
Change in reading habits	
No change	45 (9)
Read less	25 (5)
Read more	30 (6)
Years spent reading	
Below 5 years	15 (3)
Over 5 years	85 (17)
No of times reading per week	
Everyday	85 (17)
Once a week	15 (3)
Prior use of technology	
No	25 (5)
Yes	75 (15)

### Impact of Participation in eBook Clubs on Self Reported Apathy

**Figure 1.**
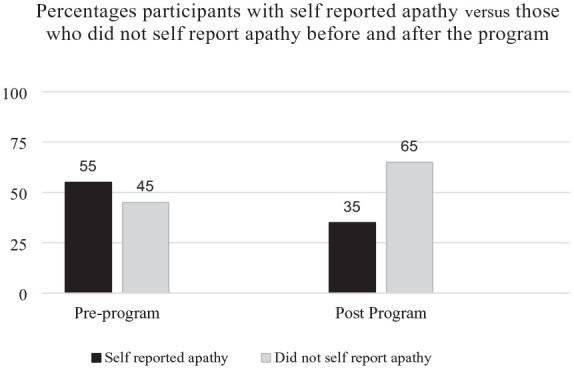
Prevalence of apathy before and after the program.

Participants reported significant social and emotional benefits from group reading sessions. Quantitative analysis revealed a significant reduction in apathy scores from baseline to post-intervention. Pre-intervention mean score: 1.6 (*SD* = 0.99); post-intervention mean score: 0.9 (*SD* = 0.71), mean difference: 0.7 (95% CI [0.23, 1.17]). A paired t-test indicated that the reduction in apathy scores was statistically significant (*t*(19) = 3.42, *p* < .01). The effect size, measured using Cohen’s d, was 0.85, indicating a large effect. Additionally, apathy prevalence decreased from 55% at baseline to 35% post-intervention (Figure 1). All participants attended at least 80% of the scheduled sessions, with an average attendance rate of 87% across the study sites.

### Key Factors Contributing to the Reduction in Apathy

Table 2 shows the joint display table of key factors contributing to the observed reduction in apathy among participants. Four major themes emerged from the qualitative data: cognitive stimulation, emotional well-being, social interaction, and program flexibility. These themes provide insight into the mechanisms underlying the reduction in apathy observed in the quantitative data.

**Table 2.** Key Factors Contributing to Apathy Reduction.

Quantitative findings	Qualitative themes	Interpretation
Apathy scores decreased from 1.6 (*SD* = 0.99) to 0.9 (*SD* = 0.71). Cohen’s *d* = 0.85 indicates a large effect.	Cognitive Stimulation: Participants found the reading material mentally engaging. Quote: “Reading keeps my brain going.”	The reduction in apathy reflects how mental engagement fostered motivation and provided participants with meaningful activity.
Proportion of participants classified as apathetic decreased from 55% to 35% after the intervention.	Emotional Well-being: Reading helped participants feel emotionally uplifted. Quote: “Even on hard days, reading gave me something to look forward to.”	Participants reported improved emotional well-being, explaining the observed decline in apathy.
87% average attendance rate across weekly sessions indicates high engagement.	Social Interaction: Group discussions fostered a sense of belonging. Quote: “Talking about books made me feel connected with others.”	Social interaction through group reading sessions contributed to reduced isolation, enhancing emotional health and engagement.
Participants reported frequent independent reading between sessions.	Program Flexibility: Participants appreciated the ability to read independently at their own pace. Quote: “I liked being able to read whenever I wanted.”	The flexibility of the eReaders encouraged independent participation, further reinforcing cognitive and emotional engagement.

### Cognitive Stimulation

Participants described the eBook club as mentally engaging, with some noting that reading kept their minds active and provided something to look forward to. This cognitive engagement aligns with the reduction in apathy scores, as it helped participants feel motivated and mentally stimulated. One participant noted:

Reading keeps my brain going; it’s like exercise for my mind. I look forward to each session, not just for the books but for the discussions. Hearing others’ thoughts and sharing my own makes me feel connected and part of something meaningful. It’s a highlight of my week, sparking curiosity and giving me a sense of accomplishment [Participant #6, 72 years]. The eReaders allowed participants to explore new topics, fostering curiosity and sustained mental engagement. Participants also reported frequent independent use of eReaders between sessions, indicating high levels of engagement with the intervention. The option to request additional books during the program further enhanced motivation and satisfaction.

### Emotional Well-Being

Participants reported that reading contributed to positive emotions and mood improvement. Engaging with new stories and ideas provided emotional relief, offering a sense of purpose and meaning. One individual stated:

Even on days I felt low, reading lifted my spirits. It gave me a sense of purpose and something positive to look forward to. The stories distracted me from negative thoughts and brought moments of joy, making my day feel brighter [Participant #4, 57 years]. Another participant reflected that engaging with new books helped them reconnect with positive emotions, contributing to improved mood. The participant commented, “It makes me laugh; even if I’m feeling sad, I can pick up a book, and everything feels lighter. The stories have a way of lifting my mood and bringing joy, turning a tough day into something much brighter” [Participant #2, 79 years].

### Social Interaction

Group discussions provided opportunities for meaningful connections with others, promoting emotional support and reducing feelings of loneliness. Participants expressed that talking about books with others made them feel part of a community, which further contributed to their emotional well-being and engagement with the program. The group sessions facilitated meaningful connections among participants. One participant shared, “I enjoyed talking about the books with others, it gave me a sense of connection and made me feel less lonely” [Participant #8, 90 years]. Sharing our thoughts and hearing different perspectives brought us closer and created a sense of community. One participant commented “There is a social aspect to it. It added to my social interaction with people. I like that” [Participant #2, 57 years]. Another participant stated, “I was able to talk to others about the books I read, which made the experience more engaging and enjoyable. It gave me a chance to connect with people and share ideas, making reading feel like a shared journey” [Participant #11, 81 years].

### Program Flexibility

Participants emphasized the importance of program flexibility in enhancing their engagement with the eBook club. Many participants appreciated the opportunity to read independently at their own pace, in addition to participating in weekly group sessions. One participant noted, “I liked that I could read whenever I wanted, without having to follow a strict schedule” [Participant #1, 84 years]. This flexibility allowed residents to integrate reading into their daily routines and provided them with a sense of autonomy. The ability to request new books throughout the program further motivated participants, as it ensured the availability of material aligned with their personal interests. For example, one participant remarked, “I was able to request books I really wanted to read, which made the experience much more enjoyable. Having a choice in what I read made it feel personalized and kept me excited about each session” [Participant #10, 75 years]. Participants also found that pre-loaded eReaders made it easier to access literature without requiring frequent technical support, further enhancing their reading experience.

## Discussion

To the best of our knowledge, this is the first study to examine the impact of eBook clubs on reducing apathy among LTCF residents. Findings indicate that group reading sessions effectively reduce apathy by combining cognitive stimulation with social interaction. Unlike interventions focused solely on individual engagement, the structured group format fosters shared experiences and emotional connections, addressing key aspects of apathy such as withdrawal and lack of motivation (Marin, 1991; Starkstein, 2000). While independent reading was available, qualitative feedback and observed outcomes highlight group dynamics as the primary driver of the intervention’s success. This is consistent with prior studies suggesting that group activities can reduce apathy in older adults (Theleritis et al., 2017; Zhu et al., 2017).

Our findings further demonstrate that eBook clubs significantly reduce self-reported apathy and enhance emotional and social well-being among LTCF residents. The reduction in apathy suggests that the interactive nature of eReaders, combined with stimulating content, actively contributes to improved health outcomes. Apathy, prevalent in LTCF, is associated with poorer cognitive and functional outcomes (Marin, 1991). By providing engaging activities, eBook clubs mitigate these risks, serving as a proactive tool for managing apathy (Oh et al., 2018; Zhu et al., 2017). Additionally, the emotional and social benefits of reading in LTCF are substantial. While traditionally seen as a solitary activity, eBook clubs foster social interaction, a critical component of emotional health being (O’Neill & Dogra, 2016). Group reading sessions encourage discussions and connections, reducing loneliness and isolation (Weziak-Bialowolska et al., 2023).

This study highlights the feasibility of eBook clubs as a practical non-pharmacological intervention for addressing apathy in LTCF residents. While music therapy reduces apathy and improves mood (Tang et al., 2018), its need for specialized training and instruments limits scalability (Cohen-Mansfield, 2018). Similarly, pet-assisted therapy provides emotional and social benefits (Bernabei et al., 2013), but logistical challenges such as animal care, allergies, and safety concerns restrict its widespread use (Zisselman et al., 1996). In contrast, eBook clubs are cost-effective, requiring only eReaders and minimal staff training, and are highly scalable. They uniquely integrate cognitive stimulation and social interaction, effectively addressing multiple dimensions of apathy while fostering emotional well-being and social connections, making them a more sustainable solution for LTCF.

Despite these potential benefits, the implementation of eReaders must be handled with sensitivity to the unique emotional and psychological needs of users. Some individuals may experience frustration or anxiety when learning new technologies, which could counteract the potential emotional benefits. Therefore, proper training and support are essential to ensure that the transition to digital reading is as smooth as possible (Mitzner et al., 2010). These may include individualized instruction (Charness & Boot, 2009), peer support (Lee & Maher, 2021), and hands-on practice (Czaja & Sharit, 2013). Moreover, care must be taken to select appropriate reading materials that are aligned with the interests and emotional states of the readers. Overly complex or emotionally distressing content could potentially exacerbate feelings of frustration or sadness.

Many participants had limited prior exposure to digital technology, contributing to difficulties in using the devices. This aligns with Andrews et al. (2019), who found that technological literacy significantly impacts older adults’ ability to use digital devices. Addressing these challenges requires targeted training tailored to their cognitive and physical needs (Wu et al., 2015). Additionally, issues such as eReader weight and navigation difficulties highlight the need for user-friendly designs suited for older adults (Kebede et al., 2022). Participants also expressed mixed preferences for digital and physical books, consistent with Lu et al. (2024), who noted older adults’ preference for the tactile experience of physical books. These findings underscore the importance of offering both formats to accommodate preferences and ensure inclusivity in eBook club programs.

The program’s implementation also revealed critical logistical considerations, such as the need for reliable Wi-Fi infrastructure when downloading books to eReaders or any digital reading devices and consistent scheduling to avoid program interruptions. These aspects are crucial for the seamless integration of technology-based interventions in LTCF (Kebede et al., 2022; Sen et al., 2022). Furthermore, the feedback from residents about the timing and accessibility of reading materials indicates the importance of aligning program logistics with residents’ daily routines and seasonal activities to maximize participation and engagement (Smith, 1993).

## Strengths and Limitations of the Study

This study addresses a critical gap in geriatric care literature by examining how technology and reading intersect to mitigate apathy in LTCF residents. However, several limitations should be noted. The small sample size of 20 participants limits the generalizability of findings, as it may not capture the diverse experiences of a larger, more varied population. Additionally, the absence of a control group or alternative conditions (e.g., independent reading) makes it difficult to isolate the specific effects of group interaction. Future studies should adopt comparative designs to better distinguish the contributions of group and individual reading.

The reliance on self-reported data collected using the GDS-3A to assess engagement and mood is another limitation. Incorporating standardized apathy measures in future research would enhance the robustness of findings. The study also did not explore how device features, such as screen glare or battery life, influence user experience, which could impact satisfaction and effectiveness. Furthermore, the role of staff and the structure of reading activities were not fully examined, despite their potential influence on outcomes. Lastly, the short follow-up period precluded an assessment of the long-term benefits of eBook clubs, highlighting the need for longer-term studies to evaluate their sustained impact on residents’ well-being.

## Implications for Practice

To boost participation in eBook clubs among LTCF residents, technology should be tailored to their needs with simplified interfaces, larger icons, high-contrast colors, and lightweight, easy-to-grip eReaders. Features such as text-to-speech and adjustable text sizes enhance accessibility (Bernard et al., 2001). Scheduling sessions strategically and offering both eReaders and physical books cater to diverse preferences, increasing satisfaction and fostering social interaction through hybrid formats. Apathy, linked to reduced neural connectivity in reward and motivation centers (Jang et al., 2021), may be counteracted by engaging stories and group discussions that stimulate these networks. Integrating eBook clubs into LTCF routines provides residents with meaningful engagement, improving quality of life.

## Conclusion

The results demonstrate the potential of eBook clubs to reduce apathy among LTCF residents. By promoting social interaction and emotional engagement, they offer a scalable and cost-effective alternative to pharmacological treatments. eBook clubs also address accessibility challenges of traditional books through eReaders with adjustable fonts and backlighting, alleviating visual strain in older adults. Additionally, eReaders provide access to extensive literary content, overcoming physical library limitations and enhancing cognitive engagement. Future research should explore the long-term effects of eBook clubs and investigate their integration with other therapeutic activities to maximize their impact on residents’ well-being.
